# Exploring the Functional Features of Melon Peel Flour for Healthier Bakery Products

**DOI:** 10.3390/foods14010040

**Published:** 2024-12-27

**Authors:** Mafalda Alexandra Silva, Tânia Gonçalves Albuquerque, Liliana Espírito Santo, Carla Motta, Agostinho Almeida, Rui Azevedo, Rita C. Alves, Maria Beatriz P. P. Oliveira, Helena S. Costa

**Affiliations:** 1 Research and Development Unit, Department of Food and Nutrition, National Institute of Health Dr. Ricardo Jorge, Avenida Padre Cruz, 1649-016 Lisbon, Portugal; mafalda.silva@insa.min-saude.pt (M.A.S.); carla.motta@insa.min-saude.pt (C.M.); helena.costa@insa.min-saude.pt (H.S.C.); 2 REQUIMTE/LAQV, Department of Chemical Sciences, Faculty of Pharmacy, University of Porto, 4050-313 Porto, Portugal; lilianaespiritosanto81@gmail.com (L.E.S.); aalmeida@ff.up.pt (A.A.); ruiazevedo43@gmail.com (R.A.); rita.c.alves@gmail.com (R.C.A.); beatoliv@ff.up.pt (M.B.P.P.O.); 3 Nutrition and Bromatology Group, Department of Analytical Chemistry and Food Science, Faculty of Science, University of Vigo, E-32004 Ourense, Spain

**Keywords:** *Cucumis melo* L. peel flour, mineral composition, sustainability, bioaccessibility, biscuit, muffins, amino acids profile

## Abstract

The use of fruit by-products to develop new food products could be an advantageous approach to meet the demand for healthy foods and reduce food waste. In this study, the amino acid and mineral profiles of melon peel flour were evaluated. Non-essential/toxic elements were also determined. Furthermore, two formulations (biscuit and muffin) were developed with 50% and 100% melon peel flour, respectively. The bioaccessibility of essential minerals in these two formulations was also determined. These innovative products presented interesting contents of amino acids and high levels of minerals, contributing significantly to daily mineral requirements, mainly magnesium (18–23%), phosphorus (13–28%), molybdenum (14–17%), and manganese (10–13%). Regarding the *in vitro* bioaccessibility of minerals in the developed formulations, magnesium, manganese, sodium, and phosphorus were those with the highest values (75–108%). Based on these results, melon peel has the potential to improve global food security, nutrition, economic well-being, and overall health and well-being.

## 1. Introduction

In the fruit industry, the post-harvest, processing, and distribution sectors generate large quantities of by-products, contributing to increased agrifood wastes in landfills. The fruit and vegetable category is one of those with the highest level of loss rate [1]. Melon (*Cucumis melo* L.) is a fruit widely consumed worldwide, and its global production generates around 8 to 20 million tons per year of by-products [2]. An alternative to this by-product is the production of melon peel flour [3,4]. These by-products are a rich source of protein, fibre, minerals, vitamins, and compounds with high antioxidant activity and beneficial properties for human health [5,6,7,8]. Many studies have been carried out on the potential of these by-products as functional ingredients, evaluating their physico-chemical composition, biological activities (antioxidant, antimicrobial, antidiabetic, among others), and techno-functional properties [9,10,11,12,13,14]. Minerals are essential for the body, performing diverse functions and being involved in various biochemical processes, such as enzymatic reactions, bone mineralisation, and hormone secretion [15]. Protein is also an essential nutrient for our body. Some amino acids are classified as essential, as humans cannot synthesise them, and their source must be the diet. Amino acids play an important role in regulating gene expression, nutrient transport and metabolism, and anti-oxidative responses, among others [16,17]. Incorporating these food by-products into food products, such as bakery products, which are generally nutritionally poor, provides multifunctionality, reducing land use and promoting the circular economy [4,18].

Recently, consumers have increased their interest in the foods they consume and their nutritional composition, quality, origin, and production methods. Consumers’ concern about the effect that some ingredients used in the formulation of food products have on their health is a reality, and therefore the interest and the search for food products without chemical additives, with a list of few ingredients, few additives or preservatives, and “clean label” products, has been increasing [19]. Gluten-free baked goods are a particularly challenging food group when it comes to formulating clean labels. Furthermore, strict gluten-free diets may not provide the necessary amounts of some nutrients, such as minerals and vitamins. Melon peel flour, as it is gluten free, could be a solution in the development of these products, trying to minimise these problems [20].

The incorporation of fruit peels in food products has been widely studied, and there are several studies on the incorporation of these by-products in bakery products, such as, incorporation of orange, passion fruit, watermelon, and melon (canary melon and muskmelon) peels in biscuits [21,22,23]; prickly pear, cajá-maga, and orange peels powders in bread [7,24,25]; grape peels in pasta formulations [26,27]; and orange passion fruit peel in cake formulations [28]. The range of food products is wide, and their use has also been studied in other types of food, such as jam [29], yogurts [30], fruit bars [31], and meat products [32,33], as well as seawater fortification with compounds from cantaloupe peels [34].

Therefore, it is important to know the nutritional composition of these by-product flours and the release of nutrients in the gastrointestinal tract since a high nutritional value does not mean that the nutrients are metabolised in the body [35]. For example, there are compounds (polyphenols, oxalates, phytates, phosphates, among others) that can inhibit the absorption of minerals, and others, such as organic acids, that increase their absorption [36,37]. *In vitro* digestion methods have been used to estimate the bioaccessibility of food nutrients, as they are more rapid, less expensive, and reproducible than in vivo methods. The bioaccessibility of a nutrient refers to the amount that is released in the gastrointestinal tract during the digestion process. The standardised INFOGEST method simulates food digestion and includes the three main stages of the human digestion procedure (oral, gastric, and intestinal phases) [38].

Therefore, this study aimed to determine the mineral (essential and non-essential/toxic elements) and amino acid profiles of melon peel flour. Furthermore, two bakery products based on melon peel flour were developed, and the bioaccessibility of the minerals was evaluated.

## 2. Materials and Methods

### 2.1. Standards and Reagents

The AccQ-Tag Chemistry kit that includes Amino Acid Hydrolysate standard, AccQFluor Borate Buffer, AccQFluor Reagent Powder; AccQFluor Reagent Diluent, AccQ-Tag ultra-eluent A; and AccQ-tag ultra-eluent B were obtained from Waters Corporation Company (Waters, Milford, MA, USA). The working solutions were prepared by diluting the standard stock solution (Waters Amino acid Hydrolysate standard) containing 2.5 mM of each amino acid (histidine (His), serine (Ser), arginine (Arg), glycine (Gly), aspartic acid (Asp), glutamic acid (Glu), threonine (Thr), alanine (Ala), proline (Pro), cysteine (Cys), lysine (Lys), tyrosine (Tyr), methionine (Met), valine (Val), isoleucine (Ile), leucine (Leu), and phenylalanine (Phe). α-Amylase (EC 232.565.6), pepsin (EC 232.629.3), bile (EC 232.369.0), and pancreatin (EC 232.468.9) were purchased from Sigma-Aldrich^®^ (St. Louis, MO, USA). Infant formula food test material was obtained from the Food Analysis Performance Assessment Scheme (FAPAS^®^) proficiency test 25254, and the test materials white cabbage (BCR-679), fish muscle (ERM-BB422), and wheat flour (ERM-BC382) were obtained from the National Institute of Standards and Technology (Gaithersburg, MD, USA). All chemicals and reagents were purchased from various commercial sources and were of analytical grade. Ultrapure water from the Milli-Q system (Millipore, Bedford, MA, USA) was used.

### 2.2. Melon Peel Flour Preparation

In 2021, the melon samples were collected from companies involved in production and distribution (Frutas A. R. Santos in Torres Vedras, Portugal and Planície Verde in Rio Maior, Portugal). The samples consisted mainly of discarded fruits that did not meet market size, shape, or colour requirements. Initially, the melons were washed with running water and then immersed in a commercialised solution of sodium hypochlorite. The peels were manually separated and cut into small pieces. The melon peels were dehydrated in a food dehydrator (Lacor 69523 Pro, Bergara, Spain) at 50 °C for 18 h after testing various temperatures and drying times to reach the desired moisture level (<10%). Subsequently, the dehydrated melon peels were blended (Grindomix GM200, Retsch, Haan, Germany) at 5000 rpm for 1 min and sieved to obtain melon peel flour. Finally, the flour was stored in a vacuum-sealed container and kept away from light.

### 2.3. Biscuits Preparation

Two types of biscuits were developed: the control and the biscuit containing 50% melon peel flour. The biscuits were prepared using wheat flour, melon peel flour, sugar, butter, and eggs. The ingredients and the respective amounts used in both biscuits are reported in a previous study [39]. Initially, the butter and sugar were mixed, and the eggs were gradually added to the mixture. Then, the flours were added, and the mixture was left to rest for 30 min. After preparing the dough, the biscuits were baked in a domestic oven (Flama 1548FL, Oliveira de Azeméis, Portugal) at 200 °C for 10 min.

### 2.4. Muffin Preparation

Two types of muffins were developed: the control muffin and the muffin with 100% melon peel flour. Several ingredients and recipes were tested to prepare these two muffins. After testing the various recipes, the best one was selected, and the muffins were prepared. The ingredients used were wheat flour, melon peel flour, bananas, soya yoghurt, eggs, dry yeast, vegetable oil, and chocolate. The ingredients and the respective amounts used in both muffins are reported in a previous study [39]. The egg yolks were added to the mixture of bananas and yogurt, previously homogenised in a food processor. Then, the dry ingredients were involved in the previous mixture. Finally, the egg whites and chocolate pieces were added. After preparing the dough, the muffins were cooked in a domestic oven (Flama 1548FL, Oliveira de Azeméis, Portugal) at 180 °C for 15 min.

### 2.5. Amino Acid Analysis

For the amino acid analysis, the pre-column derivatisation and chromatographic separation were performed according to the method described by Mota et al. (2016) [40]. For each sample, 30 mg was weighed for quartz digestion vials. A total of 200 µL of an internal standard solution (25 mM D-Norvaline) and 1 mL of hydrochloric acid (6 N) with 0.5% phenol were added. Sample digestions were performed using a closed microwave digestion system (Milestone ETHOS 1 Series, Sorisole, Italy). After hydrolysis, extracts were neutralised with 1 mL of sodium hydroxide (6 N), taken up with ultrapure water to a final volume of 10 mL, and filtered. The derivatisation process was carried out at 55 °C for 10 min after adding 80 µL of buffer and 20 µL of derivatisation reagent to 10 µL of sample in a chromatography vial.

The test material FAPAS^®^ 25254 was analysed and compared for quality assurance purposes with the assigned values.

Separation and quantification were performed on a Waters^®^ Acquity UPLC system (Waters Corporation Company, Milford, CT, USA) equipped with a photodiode array detector (PDA) and using a Acquity UPLC BEH C18 column (100 mm × 2.1 mm, 1.7 µm). The column temperature was maintained at 55 °C, and the flow rate applied was 0.7 mL/min. The mobile phase consists of two eluents: AccQTag ultra eluent A diluted in 95% ultrapure water and AccQTag ultra eluent B, both from Waters Corporation Company, Milford, CT, USA. The elution gradient followed linear staging as follows: 0–0.54 min, 0.1% B; 5.74 min, 9.1% B; 7.74 min, 21.2% B; 8.04 min, 59.6% B; 8.73–10 min, 0.1% B. The samples were monitored at 260 nm, and the injection volume was 1 µL. The peak areas were processed and quantified with Empower™ software version 2.0 (Waters, Milford, MA, USA).

### 2.6. Mineral Analysis

#### 2.6.1. Microwave-Assisted Acid Digestion

Closed-vessel acid digestion of the samples was carried out in an Ethos™ Easy microwave oven (Milestone, Sorisole, Italy) equipped with an SK-15 easyTEMP high-pressure rotor with temperature control [41]. About 400 mg of each sample was weighed into PTFE-TFM vessels, and 9 mL of concentrated nitric acid (67–69% *w*/*w*) was added. The samples were left to pre-digest in an extraction chamber for 20 min. Then, 1 mL of hydrogen peroxide (30% *w*/*w*) was added. Finally, the vessels were closed and placed in the microwave oven with the following digestion program: a gradual increase in temperature for 20 min to 210 °C, followed by 15 min at 210 °C. After cooling to room temperature, the vessels were opened in an extraction chamber and left to ventilate for approximately 10 min. Then, 0.5 mL of hydrochloric acid (>30% *w*/*w*) was added, and the solutions were transferred to decontaminated 50 mL tubes. Finally, the solutions were diluted with ultrapure water to a final volume of 50 mL. At least one blank was performed in a random vessel in each round of digestion. For quality assurance purposes, the test materials white cabbage (BCR-679), fish muscle (ERM-BB422), and wheat flour (ERM-BC382) were digested under the same conditions as the samples.

#### 2.6.2. Elemental Analysis

An inductively coupled plasma mass spectrometry (ICP-MS) iCAP™ Q instrument (Thermo Fisher Scientific, Bremen, Germany) was used for elemental analysis of the digested samples. Digested samples and blanks were diluted 10× in a diluent with nitric acid (2% *v*/*v*) and internal standard at 10 µg/L. The internal standard was prepared through the appropriate dilution of commercial single-element solutions of Ga and Rh (1000 mg/L, SCP Science, Quebec, Canada) and a multi-element solution (Internal Standard Mix 1—SCP-IS7, 10 mg/L, SCP Science). These calibration standards were diluted 10× with the same diluent used to dilute the samples. The isotopes ^25^Mg, ^31^P, ^43^Ca, ^55^Mn, ^57^Fe, ^59^Co, ^60^Ni, ^65^Cu, ^66^Zn, ^75^As, ^82^Se, ^87^Rb, ^88^Sr, ^9^8Mo, ^111^Cd, ^133^Cs, ^137^Ba, ^202^Hg, ^206^Pb, ^207^Pb, and ^208^Pb were analysed to determine the content of the samples, and the isotopes ^45^Sc, ^71^Ga, ^90^Y, ^103^Rh, ^115^In, and ^209^Bi were monitored as the internal standard. For quality assurance purposes, the test materials white cabbage (BCR-679), fish muscle (ERM-BB422), and wheat flour (ERM-BC382) were analysed under the same conditions as the samples.

### 2.7. In Vitro Digestion Model

The simulated *in vitro* digestion was performed according to the standardised static *in vitro* digestion model proposed by the COST INFOGEST network [38]. The assays were performed in triplicate for each sample. A blank tube, i.e., a tube with all enzymes, bile, and simulated digestions fluids but without sample were also performed to correct sample results. The concentration of bile salts in bile, as well as the enzymatic activities of the enzymes, were evaluated according to the Supplementary Material of the method reported by Minekus et al. (2014) [38]. The pH parameter was previously adjusted at each stage of the digestion process, using replicates of the digestion tests for each sample.

The method includes three phases: oral, gastric, and intestinal. Briefly, in the oral phase, 1 g of each sample (controls and innovative products) was mixed with 1 mL of simulated salivary fluid with α-amylase (pH 7, 75 U/mL). The tubes were placed on a mechanical shaker in an incubator at 37 °C for 2 min. For the gastric phase, 2 mL of simulated gastric fluid with pepsin (pH 3, 2000 U/mL) was added, and for the intestinal phase, 4 mL of simulated intestinal fluid with pancreatin (pH 7, 100 U/mL) and bile salts (pH 7, 10 mM). The tubes were incubated at 37 °C on a mechanical shaker for 120 min for both phases. At the end of the intestinal phase, the reaction was stopped with the addition of Pefabloc^®^ (1 mM). The tubes were placed in liquid nitrogen and kept at −80 °C until further determinations. The samples obtained at the end of the gastrointestinal simulation steps were centrifuged at 8452× *g* for 10 min at 4 °C and were subjected to the analytical procedures described in Section 2.6.1 and Section 2.6.2. to estimate the bioaccessibility values for minerals.

### 2.8. Statistical Analysis

The statistical analyses of data were performed using Microsoft Office Excel^®^ 2010 and IBM^®^ SPSS Statistics 26.0 software. Results are expressed as mean ± standard deviation (SD). For multiple comparisons of normally distributed data, parametric one-way analysis of variance (ANOVA) followed by the Tukey test was used. A level of *p* < 0.05 was considered statistically significant. Correlations between the bioaccessibility of minerals and the total mineral content were determined using Spearman’s correlation coefficients.

## 3. Results and Discussion

### 3.1. Amino Acid Composition

The amino acid profile of the melon peel flour is presented in Table 1. According to the results obtained, the essential and non-essential amino acid contents were present at 2639 and 4279 mg/100 g, respectively. The main essential amino acids in melon peel flour were leucine (458 mg/100 g) and phenylalanine (403 mg/100 g). Regarding non-essential amino acids, glutamic acid (1040 mg/100 g), aspartic acid (744 mg/100 g), and alanine (525 mg/100 g) had the highest contents in melon peel flour. Glutamic acid maintains the blood glucose level and participates in various metabolic reactions. Furthermore, it also helps to improve bowel function [42]. Leucine performs several important functions, acting as a regulator of initiating protein synthesis translation in skeletal muscle, a nitrogen donor in the production of alanine and glutamine, and a substrate for skeletal muscle [43].

Mallek-Ayadi et al. (2022) [44] also found glutamic acid, arginine, aspartic acid, and alanine to be the major amino acids present in the Maazoun melon peels. Compared with other fruit peels, melon peel flour has higher contents of histidine, isoleucine, lysine, methionine, phenylalanine, alanine, glutamic acid, serine, and tyrosine than those reported by Hussain et al. (2023) for citrus peel [45]. The same authors also analysed the amino acid profile of pomegranate and guava peels. In relation to pomegranate peel, melon peel flour has higher levels of alanine, proline, serine, and tyrosine, while compared to guava peel, it has higher levels of histidine, lysine, phenylalanine, alanine, aspartic acid, glutamic acid, and tyrosine [45]. On the other hand, melon peel flour also has higher levels of isoleucine, methionine, phenylalanine, glutamic acid, and tyrosine than the peel of citrus limetta and citrus maxima [46].

Table 2 shows the calculated amino acid scores to estimate the adequate intake for essential amino acids for adults, according to FAO (2013) [47]. The amino acid score aims to predict the quality of the protein, determining how adequate the dietary protein may be in providing the appropriate standard of essential amino acids in the diet. The amino acid score was calculated as (mg of amino acid in 1 g of test protein/mg of amino acid in requirement pattern) × 100 [47]. The essential amino acids for humans are leucine, isoleucine, valine, lysine, threonine, tryptophan, methionine, phenylalanine, and histidine. A score of 100% or higher indicates that the essential amino acid in the test protein is equal to or higher than the reference protein, and the protein can be called a complete protein; if the score is less than 100%, the test protein may be called an incomplete protein [48]. According to the results, all the scores were >100%, indicating that melon peel flour met the nutritional requirements and could be attractive as a balanced source of amino acids.

Table 1 also shows the results of the amino acid profile of the developed biscuits and muffins (controls and innovative products). The incorporation of melon peel flour allowed the development of a biscuit with an essential amino acid content of 2735 mg/100 g, with the highest levels being leucine (542 mg/100 g) and phenylalanine (438 mg/100 g). The main non-essential amino acids present in the innovative biscuit were glutamic acid (1496 mg/100 g), aspartic acid (604 mg/100 g), and serine (483 mg/100 g). The use of melon peel flour in the biscuit formulation increased the amino acid content, except for glutamic acid, proline, and leucine. The most significant increases were observed in the levels of tyrosine (120 mg/100 g) and histidine (112 mg/100 g).

The total replacement of wheat flour with melon peel flour allowed the development of a muffin with an essential amino acid content of 2166 mg/100 g. Like the innovative biscuit, the main essential amino acids in the innovative muffin were leucine (437 mg/100 g) and phenylalanine (326 mg/100 g). In the case of non-essential amino acids, the main ones were glutamic acid (839 mg/100 g) and aspartic acid (609 mg/100 g). On the other hand, unlike the innovative biscuit, the use of melon peel flour only allowed an increase in the contents of tyrosine (31 mg), cysteine (31 mg), and histidine (17 mg).

### 3.2. Mineral Composition

#### 3.2.1. Essential Elements

The results regarding the mineral composition of the melon peel flour are presented in Table 3. In total, 23 minerals were detected in the melon peel flour, 12 of which are essential elements. The total mineral content was 5046 mg/100 g. With the obtained results, it is possible to verify that the main macroelements in the melon peel flour were potassium (2576 mg/100 g) and calcium (753 mg/100 g). However, it has also interesting levels of phosphorus (629 mg/100 g) and magnesium (584 mg/100 g). These elements play vital roles in the body. Adequate potassium intake has health benefits, as potassium is associated with a reduced risk of stroke and coronary heart disease and is essential for keeping the heart and bones healthy [49]. Magnesium is involved in numerous enzymatic reactions; it is essential for vital physiological functions and has been used in treating cardiac arrhythmia, eclampsia, and pre-eclampsia [50]. In addition to its vital role in the development of bones and teeth, calcium is also involved in several essential processes, such as muscle contraction, nerve transmission, and various enzymatic reactions [51]. Like calcium, phosphorus is an essential component of bones and teeth and a substrate in crucial cellular functions [52].

Regarding the essential trace elements, the most abundant were iron (5.84 mg/100 g), manganese (2.24 mg/100 g), and zinc (1.55 mg/100 g). These essential trace elements play fundamental roles in the proper functioning of the body. Iron is part of the structure of many enzymes, and it participates in various processes such as oxygen transport; energy production; and DNA, RNA, and protein synthesis [53]. Manganese plays a vital role in bone and connective tissue growth and blood clotting, and it is a cofactor of several enzymes [54]. On the other hand, zinc plays a fundamental role in biological processes related to the organism’s growth, influencing cellular differentiation, proliferation, and apoptosis [55]. Melon peel flour can be considered a good source of essential elements due to its high content. The results obtained in this study are in accordance with those reported by Mallek-Ayadi et al. (2017) [56] for melon peel. These results also indicate that the produced melon peel flour can be nutritionally better than wheat and whole wheat flour when comparing their calcium, magnesium, phosphorus, potassium, and molybdenum contents [57]. On the other hand, melon peel flour also has much higher levels of potassium, phosphorus, and selenium than those reported for prickly pear peel flour but lower levels of magnesium, calcium, iron, manganese, and zinc [58]. Comparing the results obtained by Hadeer et al. (2023) [59] for orange, mandarin, and lemon peel flours, melon peel flour has much higher magnesium levels than those reported at 81, 70, and 92 mg/100 g, respectively. On the other hand, the levels of macroelements (magnesium, calcium, potassium, and phosphorus) and essential trace elements (iron, zinc, and copper) in melon peel flour can be considered high when compared to mango peel flour [60]. Additionally, melon peel flour has higher levels of sodium, potassium, iron, calcium, and zinc when compared to pumpkin peel flour [45].

Table 3 also shows the results of the mineral composition of the developed biscuits and muffins (controls and innovative products). The controls (biscuit and muffin) are characterised by their low levels of essential elements. The total mineral contents were 324 and 1136 mg/100 g for the control biscuit and control muffin, respectively. Concerning the developed biscuit, the incorporation of melon peel flour allowed a ~5-fold increase in their total mineral content, while its use in the developed muffin allowed an increase of approximately 1.5 times. The incorporation of melon peel flour in biscuits increased all the macroelements studied, and the highest increase was observed for potassium content (729 mg/100 g). Regarding essential trace elements, the incorporation of melon peel flour increased the content of all analysed essential trace elements, and the highest increase was observed for iron (1.5 mg/100 g). According to Regulation (EC) No. 1924/2006 on nutrition and health claims [61], the control biscuit is only considered a source of manganese, while the developed biscuit can be considered rich in potassium, phosphorus, and magnesium and a source of calcium, iron, and manganese.

Table 4 presents the dietary reference intakes for minerals according to EFSA and the respective contribution of the innovative biscuit and muffin [62]. The obtained results show that a portion of the innovative biscuit (35 g) contributes more to the recommended needs for all minerals than a portion of the control biscuit. The highest increase was observed for magnesium and calcium. A portion of the innovative biscuit can contribute 18% for adults, while the control biscuit only contributes 2% of the necessary recommendations for both minerals. The control biscuit only contributes 2% of the necessary recommendations for children, while the innovative biscuit can contribute 22%. The innovative biscuit can contribute 15%, 14%, and 10% to the recommended needs for adults for phosphorus, molybdenum, and manganese, respectively. In the case of children, the innovative biscuit contributes 17%, 13%, and 11% to the recommended needs for molybdenum, phosphorus, and manganese, respectively.

For the developed muffins, the full replacement of wheat flour with melon peel flour increased the magnesium, calcium, and potassium contents. The highest increase was observed for potassium content (374 mg/100 g). Although the phosphorus content was higher in the control muffin, no significant differences (*p* > 0.05) were found. Regarding essential trace elements, the incorporation of melon peel flour increased the content of iron, manganese, cobalt, and molybdenum, and the highest increase was also observed for iron content (0.5 mg/100 g). The copper, zinc, and selenium contents were higher in the control muffin; however, no significant differences (*p* > 0.05) were found. According to the same regulation, the control muffin can be considered a source of potassium and manganese and rich in copper and phosphorus, while the muffins developed are rich in potassium, phosphorus, and magnesium and a source of calcium, iron, manganese, and copper. Regarding the contribution of muffins to the recommended mineral needs, the highest increase was observed for magnesium (Table 4). A portion of the innovative muffin (48 g) can contribute 19%, while the control muffin only contributes 6% of the necessary recommendations for magnesium. On the other hand, the control muffin only contributes 7% of the necessary recommendations for children, while the innovative muffin can contribute 23%. For adults, the innovative muffin can contribute 14%, 11%, and 10% of the recommended needs for molybdenum, manganese, and potassium, respectively. For children, a portion of the innovative muffin can contribute 17%, 13%, and 11% of the recommended needs for molybdenum, manganese, and potassium, respectively. Regarding phosphorus, both muffins contribute equally to the recommended needs, 28% and 24% for adults and children, respectively.

#### 3.2.2. Non-Essential and Toxic Trace Elements

In this study, 11 non-essential elements were detected in the melon peel flour (Table 5). The most abundant non-essential and toxic trace elements in the melon peel flour were aluminium (2692 µg/100 g), strontium (2070 µg/100 g), and rubidium (1954 µg/100 g). Mercury (1.2 µg/100 g) and titanium (2.7 µg/100 g) were the least abundant. Beryllium, arsenic, and antimony contents were below their respective LOD, with arsenic being one of the four major elements of toxicological concern. Regarding the innovative biscuit, the least abundant were titanium (0.81 µg/100 g), mercury (1.8 µg/100 g), and lead (2.1 µg/ 100 g), and the most abundant were aluminium (985 µg/100 g), strontium (631 µg/100 g), and rubidium (621 µg/100 g). For the innovative muffin, the least abundant were titanium (0.47 µg/100 g), antimony (1.2 µg/100 g), and caesium (1.7 µg/ 100 g), and the most abundant were aluminium (975 µg/100 g), rubidium (544 µg/100 g), and strontium (411 µg/100 g). It should be highlighted that the arsenic and mercury contents of the innovative muffin were below their respective LODs.

Interest in the use of fruit by-products by various industries has been increasing. However, besides compounds beneficial to health, these by-products can also contain toxic compounds. The accumulation of toxic trace elements has adverse effects on our health, which can lead to the development of several severe diseases in various organs of the human body [63,64]. Although there are maximum levels set for certain toxic trace elements for some foodstuffs, namely, lead (0.010–3 mg/kg), cadmium (0.005–3 mg/kg), and mercury (0.10–1 mg/kg), there are still no maximum levels set for this type of flour [65]. Therefore, it becomes increasingly important to characterise its safety profile for both the consumer and the environment.

### 3.3. In Vitro Bioaccessibility of the Elements in the Biscuits and Muffins

Determining the total amount of minerals present in the analysed flours is very important; however, these values may not represent the benefits that the human diet provides. As a result, studies focusing on bioaccessibility are useful because they can reveal which food matrices are most suitable for obtaining the levels of micronutrients that the body can absorb [66]. Bioaccessibility refers to the fraction of a given nutrient released from the food matrix in the gastrointestinal tract, becoming soluble and available to be absorbed [38]. The bioaccessibility of minerals was calculated using the amount of minerals in the supernatant obtained at the end of the intestinal phase and the amount of minerals in the sample before digestion. Table 6 presents the bioaccessibility values obtained for magnesium, phosphorus, calcium, sodium, potassium, manganese, iron, copper, cobalt, and molybdenum in the developed biscuits and muffins (control and innovative products). In the case of biscuits, the bioaccessibility values of magnesium, copper, and molybdenum were higher for the innovative product, while the bioaccessibility values of calcium, potassium, and manganese were higher in the control product. Molybdenum and calcium showed the lowest bioaccessibility values for the control biscuit and innovative biscuit, respectively.

The bioaccessibility value obtained for magnesium was the same in both muffins, while the control muffin showed higher bioaccessibility values for the remaining minerals. In the case of the control muffin, phosphorus, molybdenum, sodium, and manganese were the minerals that presented higher bioaccessibility values, while for the innovative muffin, the minerals with the highest values were magnesium, phosphorus, and potassium. On the other hand, calcium had the lowest bioaccessibility in the two products.

The relationship between the bioaccessibility of minerals and the total mineral content was assessed using Spearman’s correlation (Table 7). The bioaccessible fraction of magnesium showed a positive correlation with all minerals, showing a strong positive correlation with the levels of calcium, potassium, manganese, and molybdenum (r = 0.949). The bioaccessible fractions of cobalt and molybdenum also showed a positive correlation with all minerals. On the other hand, the bioaccessible fraction of calcium showed a negative correlation with the levels of all minerals. The bioaccessible fractions of phosphorus and sodium were negatively affected by the magnesium, calcium, potassium, manganese, and molybdenum contents (r = −0.105) and showed a positive correlation with the copper and cobalt contents (r = 0.949 and r = 0.738, respectively).

Components such as tannins, phytates, and oxalates, present in melon peel flour, can negatively affect the bioaccessibility of some minerals [66,67]. Ferreira and Tarley (2020) [66] found a negative correlation between calcium and the presence of phytic acid. Many authors have reported low calcium bioaccessibility values in different fruit flours [35,66,67]. Calcium bioaccessibility can be negatively affected by some components present in foods, such as tannins, phytates, oxalates, phosphates, and fibre. These components can have an inhibitory effect, forming insoluble complexes or through precipitation, creating insoluble salts [35,68]. This may explain the low calcium bioaccessibility observed in developed foods. Phytic acid can strongly bind to calcium and other divalent mineral cations, such as magnesium, iron, and zinc, decreasing their bioaccessibility [69]. Galvão et al. (2023) [70] observed a negative correlation between the bioaccessible fraction of calcium and ash content. The same authors also found a negative correlation between the bioaccessible fraction of copper and the content of total phenolic compounds. These compounds can form insoluble complexes with metal cations, reducing the bioaccessibility of elements such as iron, zinc, and copper. The oxidation state of iron can affect its bioaccessibility. Non-haem iron (Fe^3+^), which is the predominant form present in fruits and vegetables, has lower absorption, due to its low solubility in intestinal pH, than haem iron (Fe^2+^). Low iron bioaccessibility values can also be explained by the formation of insoluble complexes with polyphenols and antinutrients [66,71]. Ferreira and Tarley (2020) [66] observed a negative correlation between copper and phytic acid content in green banana flour. This may have been due to the formation of ternary complexes of polyvalent cations with proteins and phytic acid. On the other hand, Galvão et al. (2023) [70] reported that magnesium bioaccessibility can be influenced by total dietary fibre and fat content, as well as acidity. The presence of negatively charged polysaccharides can lead to the formation of complexes, while unsaturated fatty acids can form insoluble salts binding to bivalent metals [72].

## 4. Conclusions

Melon peel flour has interesting levels of amino acids and can be considered a complete source of essential amino acids. On the other hand, it can also be considered a good source of macroelements such as potassium, calcium, and phosphorus, and essential trace elements such as iron, manganese, and zinc. The use of high quantities of melon peel flour allowed the development of two food products (biscuit and muffin) with high levels of minerals and amino acids, contributing to their nutritional enrichment. Additionally, according to our previous study, the two food products were well accepted by an untrained tasting panel. Overall, both the biscuit containing 50% melon peel flour and the muffin with 100% melon peel flour were well accepted by the tasters, obtaining scores of 6 and 5 for overall acceptability on a seven-point hedonic scale. Furthermore, consuming a portion of the biscuit and muffin contributes to the daily mineral requirements, especially for magnesium, phosphorus, molybdenum, and manganese. Regarding the assessment of the bioaccessibility of minerals in the developed foods, magnesium, manganese, sodium, and phosphorus were the minerals with the highest values, while calcium and copper presented the lowest values. Furthermore, the use and valorisation of these by-products are very important aspects of improving food security and nutritional well-being on a global scale. Reducing and managing food waste contributes to more sustainable production in the food industry. As part of a circular economy strategy, reducing food waste directly impacts the environment (reduction of pollution) and the economy (economic valorisation of by-products) and meets the United Nations Sustainable Development Goals.

## Figures and Tables

**Table 1:** Amino acid composition (mg/100 g) of the melon peel flour, biscuits, and muffins.

Amino Acid	Melon Peel Flour	Biscuit		Muffin	
		Control	Innovative	Control	Innovative
Essential					
Histidine	268 ± 13.2	154 ± 0.8 ^a^	266 ± 2.9 ^b^	160 ± 8.2 ^A^	177 ± 0.4 ^A^
Isoleucine	293 ± 20.7	310 ± 0.7 ^a^	315 ± 0.6 ^b^	294 ± 1.9 ^A^	248 ± 1.4 ^B^
Leucine	458 ± 27.6	602 ± 1.4 ^a^	542 ± 1.7 ^b^	564 ± 4.4 ^A^	437 ± 2.6 ^B^
Lysine	367 ± 17.4	276 ± 13.1 ^a^	301 ± 8.0 ^a^	346 ± 19.4 ^A^	304 ± 6.9 ^A^
Methionine	214 ± 9.2	153 ± 5.8 ^a^	214 ± 5.7 ^b^	157 ± 6.2 ^A^	138 ± 2.4 ^A^
Phenylalanine	403 ± 24.0	403 ± 4.8 ^a^	438 ± 3.4 ^b^	346 ± 19.1 ^A^	326 ± 2.3 ^A^
Threonine	276 ± 21.6	250 ± 3.8 ^a^	287 ± 0.4 ^b^	272 ± 8.5 ^A^	232 ± 2.5 ^A^
Valine	360 ± 24.6	372 ± 0.2 ^a^	372 ± 0.1 ^b^	381 ± 5.3 ^A^	304 ± 0.5 ^B^
Non-essential					
Alanine	525 ± 36.0	322 ± 7.3 ^a^	384 ± 0.5 ^a^	346 ± 3.1 ^A^	310 ± 3.1 ^B^
Arginine	419 ± 65.9	317 ± 5.5 ^a^	354 ± 0.4 ^a^	327 ± 12.8 ^A^	315 ± 3.8 ^A^
Aspartic acid	744 ± 60.4	537 ± 36.5 ^a^	604 ± 0.7 ^a^	688 ± 17.9 ^A^	609 ± 23.6 ^A^
Cysteine	n.d.	53.7 ± 9.9 ^a^	150 ± 3.0 ^b^	40.3 ± 4.0 ^A^	71.4 ± 1.5 ^B^
Glutamic acid	1040 ± 135.2	2289 ± 99.4 ^a^	1496 ± 13.2 ^a^	1544 ± 23.5 ^A^	839 ± 30.4 ^B^
Glycine	360 ± 24.9	267 ± 3.3 ^a^	286 ± 5.3 ^a^	255 ± 9.8 ^A^	229 ± 3.5 ^A^
Proline	348 ± 14.0	698 ± 1.5 ^a^	481 ± 4.7 ^b^	462 ± 2.4 ^A^	260 ± 1.7 ^B^
Serine	427 ± 28.0	453 ± 5.4 ^a^	483 ± 11.6 ^a^	452 ± 8.4 ^A^	366 ± 5.3 ^B^
Tyrosine	416 ± 31.5	270 ± 5.9 ^a^	390 ± 1.6 ^b^	239 ± 14.3 ^A^	270 ± 2.7 ^A^

n.d., not detected. Values are average of three individual samples (*n* = 3), expressed as mean ± standard deviation. Mean values with different superscript lowercase letter and different superscript capital letter within a row are significantly different (*p* < 0.05).

**Table 2:** Amino acid score according to the FAO requirement pattern for adults (mg/g protein) of the melon peel flour.

Amino Acid	Requirement Pattern (mg/g Protein) ^1^	Amino Acid Score (%) ^2^
Histidine	15	258 ± 13
Isoleucine	30	141 ± 10
Leucine	59	112 ± 7
Lysine	45	118 ± 6
Threonine	23	174 ± 14
Valine	39	134 ± 9
Total sulphur amino acids (Met + Cys)	22	141 ± 6
Total aromatic amino acids (Phe + Tyr)	38	312 ± 21

^1^ FAO, 2013 [23]. ^2^ Amino acid score = (mg of amino acid in 1 g of test protein/mg of amino acid in requirement pattern) × 100.

**Table 3:** Essential elements of the melon peel flour, biscuits, and muffins.

Elements	Melon Peel Flour	Biscuit		Muffin	
		Control	Innovative	Control	Innovative
Macroelements					
Magnesium (mg/100 g)	584 ± 13	15.1 ± 0 ^a^	170 ± 4 ^b^	40.7 ± 1 ^A^	129 ± 3 ^B^
Phosphorus (mg/100 g)	629 ± 11	96.5 ± 2 ^a^	243 ± 4 ^b^	326 ± 5 ^A^	321 ± 8 ^A^
Calcium (mg/100 g)	753 ± 6	24.8 ± 0 ^a^	226 ± 3 ^b^	33.6 ± 0 ^A^	143 ± 0 ^B^
Sodium (mg/100 g)	487 ± 32	45.0 ± 1 ^a^	192 ± 15 ^b^	385 ± 4 ^A^	343 ± 5 ^B^
Potassium (mg/100 g)	2576 ± 67	141 ± 1 ^a^	870 ± 42 ^b^	347 ± 6 ^A^	721 ± 9 ^B^
Essential trace elements					
Manganese (mg/100 g)	2.24 ± 0.1	0.339 ± 0.0 ^a^	0.817 ± 0.0 ^b^	0.418 ± 0.0 ^A^	0.676 ± 0.0 ^B^
Iron (mg/100 g)	5.84 ± 0.3	0.686 ± 0.0 ^a^	2.21 ± 0.1 ^b^	2.14 ± 0.4 ^A^	2.65 ± 0.1 ^A^
Cobalt (µg/100 g)	10 ± 2.11	<LOD ^a^	3.2 ± 0.29 ^b^	3.9 ± 0.08 ^A^	4.3 ± 0.19 ^A^
Copper (mg/100 g)	0.332 ± 0.0	0.0873 ± 0.0 ^a^	0.137 ± 0.0 ^b^	0.322 ± 0.0 ^A^	0.281 ± 0.0 ^A^
Zinc (mg/100 g)	1.55 ± 0.1	0.472 ± 0.0 ^a^	0.807 ± 0.1 ^b^	0.946 ± 0.1 ^A^	0.928 ± 0.1 ^A^
Selenium (µg/100 g)	6.9 ± 0.67	<LOD ^a^	9.1 ± 1.23 ^b^	12 ± 1.55 ^A^	9.9 ± 0.04 ^A^
Molybdenum (µg/100 g)	78 ± 3.26	6.7 ± 0.28 ^a^	27 ± 2.29 ^b^	8.8 ± 0.14 ^A^	19 ± 0.41 ^B^

Values are average of three individual samples (*n* = 3), expressed as mean ± standard deviation. Means values with different superscript lowercase letter and different superscript capital letter within a row are significantly different (*p* < 0.05). LOD, limit of detection.

**Table 4:** Dietary reference intake for minerals, adults and children (12–17 years), and the respective contribution (%) of the consumption of biscuits and muffins (per serving).

Elements	Dietary Reference Intake		Estimated Daily Intake (%)							
			Biscuit				Muffin			
			Control		Innovative		Control		Innovative	
	Adults	Children	Adults	Children	Adults	Children	Adults	Children	Adults	Children
Calcium (mg/day)	975 **	**1150**	2	2	18	22	2	1	7	6
Copper (mg/day)	1.5 *	1.2 *	2	3	3	4	10	13	9	11
Iron (mg/day)	13.5 *	12 *	2	2	6	6	8	9	9	11
Magnesium (mg/day)	325 *	250 *	2	2	18	22	6	7	19	23
Manganese (mg/day)	3	2.5 **	4	5	10	11	7	8	11	13
Molybdenum (µg/day)	65	55	4	4	14	17	7	8	14	17
Phosphorus (mg/day)	550	640	6	5	15	13	28	24	28	24
Potassium (mg/day	3500	3100 **	1	2	9	10	5	5	10	11
Selenium (µg/day)	70	63 **	-	-	5	5	8	9	7	8
Zinc (mg/day)	11.5 *	10 **	1	2	2	3	4	4	4	4

Values in bold refer to population reference intake (PRI). * Average adequate intake values for men and women. ** Average adequate intake values for children (12–17 years).

**Table 5:** Non-essential trace elements (µg/100 g of sample) of the melon peel flour, biscuits, and muffins.

Non-Essential Trace Elements	Melon Peel Flour	Biscuit		Muffin	
		Control	Innovative	Control	Innovative
Lithium	15 ± 1.99	<LOD ^a^	4.7 ± 0.69 ^b^	5.6 ± 0.05 ^A^	3.4 ± 0.89 ^B^
Beryllium	<LOD	<LOD	<LOD	<LOD	<LOD
Aluminium	2692 ± 249	314 ± 18 ^a^	985 ± 59 ^b^	643 ± 53 ^A^	975 ± 11 ^A^
Nickel	67 ± 4.86	7.7 ± 2.78 ^a^	26 ± 2.99 ^b^	56 ± 2.18 ^A^	53 ± 1.89 ^A^
Arsenic	<LOD	<LOD	<LOD	<LOD	<LOD
Rubidium	1954 ± 55	104 ± 1.66 ^a^	621 ± 9.26 ^b^	296 ± 3.87 ^A^	544 ± 7.42 ^B^
Strontium	2070 ± 65	62 ± 2.11 ^a^	631 ± 18 ^b^	119 ± 1.10 ^A^	411 ± 5.40 ^B^
Cadmium	9.0 ± 0.06	0.71 ± 0.05 ^a^	3.1 ± 0.35 ^b^	1.2 ± 0.04 ^A^	2.2 ± 0.06 ^B^
Antimony	<LOD	<LOD	<LOD	1.8 ± 0.12 ^A^	1.2 ± 0.15 ^B^
Caesium	8.7 ± 0.57	0.18 ± 0.03 ^a^	2.4 ± 0.15 ^b^	0.48 ± 0.04 ^A^	1.7 ± 0.13 ^B^
Barium	771 ± 27	55 ± 0.82 ^a^	240 ± 7.21 ^b^	96 ± 2.78 ^A^	184 ± 6.73 ^B^
Mercury	1.2 ± 0.08	1.2 ± 0.01 ^a^	1.8 ± 0.01 ^b^	<LOD	<LOD
Titanium	2.7 ± 0.23	<LOD ^a^	0.81 ± 0.18 ^b^	<LOD ^A^	0.47 ± 0.04 ^B^
Lead	5.6 ± 0.20	<LOD ^a^	2.1 ± 0.13 ^b^	5.8 ± 0.54 ^A^	4.2 ± 0.43 ^B^

Values are average of three individual samples (*n* = 3), expressed as mean ± standard deviation. Means values with different superscript lowercase letter and different superscript capital letter within a row are significantly different (*p* < 0.05). LOD, limit of detection.

**Table 6:** Bioaccessibility (%) of essential minerals in biscuits and muffins.

Elements	Biscuit		Muffin	
	Control	Innovative	Control	Innovative
Magnesium	66 ± 7.7 ^a^	102 ± 1.3 ^a^	85 ± 2.6 ^A^	85 ± 4.1 ^A^
Phosphorus	n.d.	n.d.	105 ± 1.8 ^A^	84 ± 4.9 ^B^
Calcium	73 ± 12.9 ^a^	50 ± 1.1 ^b^	54 ± 0.7 ^A^	44 ± 2.2 ^B^
Sodium	n.d.	n.d.	95 ± 17.1 ^A^	79 ± 4.0 ^A^
Potassium	96 ± 11.3 ^a^	79 ± 5.6 ^a^	91 ± 4.4 ^A^	83 ± 5.5 ^A^
Manganese	108 ± 2.3 ^a^	101 ± 0.5 ^b^	95 ± 2.2 ^A^	52 ± 1.5 ^B^
Iron	n.d.	n.d.	83 ± 0.8 ^A^	52 ± 6.2 ^B^
Cobalt	(a)	99 ± 6.3	61 ± 2.3 ^A^	56 ± 8.0 ^A^
Copper	65 ± 1.6 ^a^	85 ± 1.4 ^b^	62 ± 5.7 ^A^	54 ± 1.7 ^A^
Molybdenum	52 ± 11.0 ^a^	88 ± 8.0 ^b^	96 ± 9.7 ^A^	66 ± 2.0 ^B^

n.d., not determined; (a) the cobalt content of the control biscuit was lower than the limit of detection. Values are average of three individual samples (*n* = 3), expressed as mean ± standard deviation. Means values with different superscript lowercase letter and different superscript capital letter within a row are significantly different (*p* < 0.05).

**Table 7:** Correlation matrix between the bioaccessibility of minerals and the total mineral content.

	TMg	TP	TCa	TNa	TK	TMn	TFe	TCo	TCu	TMo
Bio-Mg	0.949	0.316	0.949	0.316	0.949	0.949	0.632	0.316	0.316	0.949
Bio-P	−0.105	0.949	−0.105	0.949	−0.105	−0.105	0.211	0.738	0.949	−0.105
Bio-Ca	−0.800	−0.400	−0.800	−0.400	−0.800	−0.800	−1.000 **	−0.800	−0.400	−0.800
Bio-Na	−0.105	0.949	−0.105	0.949	−0.105	−0.105	0.211	0.738	0.949	−0.105
Bio-K	−1.000 **	−0.200	−1.000 **	−0.200	−1.000 **	−1.000 **	−0.800	−0.400	−0.200	−1.000 **
Bio-Mn	−0.400	−0.800	−0.400	−0.800	−0.400	−0.400	−0.800	−1.000 **	−0.800	−0.400
Bio-Fe	−0.105	0.949	−0.105	0.949	−0.105	−0.105	0.211	0.738	0.949	−0.105
Bio-Co	0.800	0.400	0.800	0.400	0.800	0.800	0.400	0.200	0.400	0.800
Bio-Cu	0.200	−0.600	0.200	−0.600	0.200	0.200	−0.400	−0.800	−0.600	0.200
Bio-Mo	0.400	0.800	0.400	0.800	0.400	0.400	0.200	0.400	0.800	0.400

T, Total mineral content; Bio, Bioaccessible fraction. ** *p* < 0.01.

## Data Availability

The original contributions presented in this study are included in the article. Further inquiries can be directed to the corresponding author.
